# Cell Proliferation to Evaluate Preliminarily the Presence of Enduring Self-Regenerative Antioxidant Activity in Cerium Doped Bioactive Glasses

**DOI:** 10.3390/ma13102297

**Published:** 2020-05-15

**Authors:** Alexandre Anesi, Gianluca Malavasi, Luigi Chiarini, Roberta Salvatori, Gigliola Lusvardi

**Affiliations:** 1 SMECHIMAI, Università di Modena e Reggio Emilia, Largo del Pozzo 71, 41125 Modena, Italy; alexandre.anesi@unimore.it (A.A.); luigi.chiarini@unimore.it (L.C.); roberta.salvatori@unimore.it (R.S.); 2 DSCG, Università degli Studi di Modena e Reggio Emilia, via G. Campi 103, 41125 Modena, Italy; gianluca.malavasi@unimore.it

**Keywords:** antioxidant activity, self-regeneration, bioactive glasses, cerium, cerium nanoparticles

## Abstract

(1) Background: a cell evaluation focused to verify the self-regenerative antioxidant activity is performed on cerium doped bioactive glasses. (2) Methods: the glasses based on 45S5 Bioglass^®^, are doped with 1.2 mol%, 3.6 mol% and 5.3 mol% of CeO_2_ and possess a polyhedral shape (~500 µm^2^). Glasses with this composition inhibit oxidative stress by mimicking catalase enzyme (CAT) and superoxide dismutase (SOD) activities; moreover, our previous cytocompatibility tests (neutral red (NR), 3-(4,5-Dimethylthiazol-2-yl)-2,5-diphenyltetrazolium bromide (MTT) and Bromo-2-deoxyUridine (BrdU)) reveal that the presence of cerium promotes the absorption and vitality of the cells. The same cytocompatibility tests were performed and repeated, in two different periods (named first and second use), separated from each other by four months. (3) Results: in the first and second use, NR tests indicate that the presence of cerium promotes once again cell uptake and viability, especially after 72 h. A decrease in cell proliferation it is observed after MTT and BrdU tests only in the second use. These findings are supported by statistically significant results (4) Conclusions: these glasses show enhanced proliferation, both in the short and in the long term, and for the first time such large dimensions are studied for this kind of study. A future prospective is the implantation of these bioactive glasses as bone substitute in animal models.

## 1. Introduction

Free radicals are formed during all the enzymatic reactions that require electron transfer, especially during the oxidative phosphorylation that occurs in cellular respiration.

Between free radicals, reactive oxygen species (ROS) are the most frequently formed and widespread: they have high reactivity, caused by the need to bring their outermost shell of the atom to a stable configuration with a damaging oxidative effect on the cell molecules of the organism.

Free radicals produced in the chain of radical reactions cause prevalent damage to phospholipids and polyunsaturated fatty acids present in the membranes of plasma and cellular organelles. The peroxidative damage is achieved through the complete destabilization of the lipid and protein structures, with the formation of insoluble and nonfunctional polymers [1,2]. ROS cause oxidation of lipids, nucleic acids and proteins; ROS damages are an underlying basis of several diseases (e.g., cancer, inflammatory and neurodegenerative diseases) [3].

The ROS scavenging system is the defense mechanisms that our body uses to protect itself from the formation of free radicals; it can be divided into enzymatic and nonenzymatic mechanisms. Among the enzymatic defence of the human organism, we include the superoxide dismutase (SOD), the catalase enzyme (CAT), especially present in cellular peroxisomes, and the oxidase (OXI) [4,5,6]. Nonenzymatic mechanisms comprise ascorbate (Vit C), glutathione, tocopherol (Vit E), polyphenols, alkaloids and carotenoids [7].

Excellent catalytic activities are exhibited by nanoceria (cerium nanoparticles, CeO_2_NPs) that are becoming more and more of medical interest. CeO_2_NPs protect cells against ROS-induced damage, exhibit redox state-dependent catalase mimetic [8,9] and neuroprotective activities [10]. The anticancer effect of CeO_2_NPs is also studied on human colorectal, lung, breast, ovarian cancer cell lines [11,12]. Pharmacological application concerning to the self-regenerating antioxidant activity of CeO_2_NPs are reported [12].

Cerium compounds are particularly interesting for pharmacological properties and are used as antibiotics, bacteriostatics, immunomodulatory agents in degenerative pathologies and as antitumorals [11,12,13,14,15,16,17,18,19,20,21,22].

In the field of biomaterials, one of the most studied bioglasses is the 45S5Bioglass^®^ [23,24]. It is constituted of 45 wt% SiO_2_, 24.5 wt% Na_2_O, 24.5 wt% CaO and 6 wt% P_2_O_5_; high amounts of Na_2_O and CaO and a very high CaO/P_2_O_5_ ratio make its surface very reactive in a physiological environment, forming a chemical bond with living tissue and stimulating healing [25,26]. Its first clinical use in an implant was aimed to replace small bones in the middle ear to treat conductive hearing loss [27]. Since then, the possible applications in the biomedical field have been widely studied [28,29,30,31,32,33,34,35,36,37].

Since its discovery, changes in the composition of the 45S5Bioglass^®^ to develop materials with increasingly bioactive characteristics have followed, such as the addition of gallium, zinc, magnesium and strontium to improve the formation of new bone [22,38,39,40].

Our attention was especially focused to develop cerium doped bioactive glasses able to prevent oxidative stress after implantation in bone, with a consequent reduction of reparative osteogenesis time and bone healing [17,18]. Natural human bone is a mineralized collagen-based connective tissue, with specific cell types, i.e. osteoblasts, osteocytes and osteoclasts [41].

To obtain bone substitutes with tailored properties and selected applications, we have studied bioactive glasses characterized by different compositions, synthesis conditions and dimensions (powders of micro and meso dimensions, polygonal slices or polyhedrons). 

We have also demonstrated [17,18,19] that i) the simultaneous presence of phosphate and cerium in the bulk or on the surface of the glasses are associated to their chemical durability; ii) the interconversion between Ce^3+^ and Ce^4+^ catalyzes the reduction of H_2_O_2_ and O_2_^−^ concentrations mimicking the CAT and SOD activities. 

In CeO_2_NPs and cerium doped bioactive glasses, the antioxidant activity, derived from quick interconversion between the oxidation states Ce^4+^ and Ce^3+^, may lead to a reversible oxidation–reduction reaction and to a distinguishing self-yield of the regenerative antioxidant activity not present in trivial biological antioxidants [20,42,43]. Few studies have investigated the potential self-regenerating antioxidant activity in CeO_2_NPs [42,43,44,45,46,47] and none in cerium doped bioactive glasses.

Recently, we have studied the biocompatibility of 45S5Bioglass^®^ doped with an increasing amount of CeO_2_ (1.2 mol%, 3.6 mol% and 5.3 mol%), soaked in (Dulbecco’s Modified Eagle Medium) DMEM for different times and tested in a cell culture medium with osteocyte-like murine long bone (MLO-Y4) and mouse embryonic fibroblast (NIH/3T3) cell lines [48]; these cellular types are particularly informative for investigation on bone substitutes [49]. 

Glasses with 1.2 mol% and 3.6 mol% of CeO_2_ show a good bioactivity (formation of hydroxyapatite, HA) and promote cell uptake and viability, favouring also cell proliferation in the case of 3.6 mol% of CeO_2_. Highest cerium content (5.3 mol%) promotes cell proliferation, but inhibits the HA formation; the increased cerium content favours high cerium ion release in DMEM and a consequent formation of an insoluble competitive CePO_4_ crystalline phase.

On the basis of these last findings and our previous results related to the antioxidant activities, here we investigate these doped glasses based on 45S5Bioglass^®^ by means of cytocompatibility tests (repeated twice,) in order to verify if the proliferation enhancement persists over time. 

## 2. Materials and Methods 

### 2.1. Synthesis of Glasses

The glass samples were prepared by the melting method [18] and from a mixture of precursors: SiO_2_, Na_2_CO_3_, CaCO_3_, Na_3_PO_4_·12H_2_O and CeO_2_ in about a total of 100 g. The powders were melted in a platinum crucible at 1350 °C using two heating rates: 5 °C/min in the range 20–1000 °C and 15 °C/min above 1000 °C. The melt was refined for 2 h at the melting temperature, then quenched on a graphite plate mold to obtain a polyhedral shape (total surface of ~500 µm^2^). The obtained samples were successively annealed at 400 °C for 2 h in order to reduce internal stress. 

The parent glass (BG) and the molar compositions of the cerium-containing glasses (BG_1.2, BG_3.6 and BG_5.3) are reported in Table 1. 

The compositions of the glasses were verified by means of elemental analysis (ICP-OES: inductively coupled plasma optical emission spectrometry).

### 2.2. Time Interval for the Execution of the Assays

The samples were utilized twice in cytocompatibility tests, in two different periods.

First use and second use (repeated after four months). This “repeated use methodology” is the in vitro cell verification of enduring protective activity of cerium doped glasses against metabolic wastes of cells maintained in culture.

Cytocompatibility tests (neutral red (NR) uptake and 3-(4,5-Dimethylthiazol-2-yl)-2,5-diphenyltetrazolium bromide (MTT) test and Bromo-2-deoxyUridine (BrdU)] were performed twice, in the same conditions on the same samples. 

After the first use, every sample was removed from the well, washed twice with Dulbecco’s-Phosphate Buffer Solution (D-PBS) (Euroclone, Milan, Italy) and dried in an oven at 37 °C to eliminate excess of water. 

All samples were bagged, tagged, and sterilized by autoclaving at 121 °C for 20 min; then stored in a dry, dark and cool place. Cell lines used do not require ethical approval, because they are cell lines already ethically approved in the ISO EN 10993-5, as standard used to perform the cell test here described.

### 2.3. Assessment of Cytocompatibility

In accordance to “Biological evaluation of medical devices — Part 5: Tests for in vitro cytotoxicity” (ISO 10993-5:) [49], osteocyte-like cell line murine long bone (MLO-Y4) was chosen for biological tests, because this line belongs to primary cell types of bone, target tissue for our bioactive glasses [30,33,50,51]. 

This cell line was grown in Dulbecco’s modified Eagle’s medium (DMEM) (Euroclone, Milan, Italy) 0.01 M (pH 7.4 phosphate buffer saline, without calcium and magnesium ions), supplemented with 10% fetal bovine serum (Invitrogen-Thermo Fisher Scientific Corporation, Whaltam, MA, USA), 100 μg/mL pen-streptomycin (Euroclone, Milan, Italy), D-Glutamine 2 mM and sodium pyruvate 1 mM (Euroclone, Milan, Italy), at 37 °C in a humidified atmosphere of 5% CO_2_ in air.

All samples underwent both direct (NR uptake) and indirect (extract, eluate) contacts for the cytocompatibility assays (MTT and BrdU, see below) [29]. 

Positive and negative control material (CTRL+ and CTRL−) were executed in accordance to part 5 of ISO-10993 [49].

For the MTT and BrdU tests, the samples were immersed in DMEM (Euroclone, Milan, Italy) without fetal bovine serum (FBS) at 37 °C for 72 h. The ratio between glass and DMEM (0.2g/mL) was chosen in accordance with the cellular test conditions proposed in the ISO 10993-12 method [52]. Since several microorganisms experienced optimum growth at 37 °C, the eluate was filtered using a 0.22 μm filter (Merck Millipore, Darmstadt, Germany) as an additional security procedure to ensure elimination of potential microbial species and make the eluate appropriate before incubation with cells [52,53].

#### 2.3.1. Direct Viability Test: Neutral Red Uptake (NR) 

NR uptake (NR solution N2889 Sigma-Merck, Darmstadt, Germany) is a common parameter of cytotoxicity, widely employed to assess the number of viable cells in a culture [54]. NR is a vital dye which concentrates on lysosomes of viable cells. Cytotoxicity is calculated as a decrease in the NR uptake into the cell after 24 and 72 h of direct exposure to the material. Cells (3 × 10^5^/mL_DMEM_ for tests at 24 h and 1.5 × 10^5^/mL_DMEM_ for tests at 72 h) were seeded in a multiwell culture plate (six wells) and maintained in culture with the glasses, at 37 °C ± 1 °C, 90% ± 5% humidity and 5.0% ± 1% CO_2_/air.

300 μL of NR solution was added after removing culture medium on the well for 3h. NR solution was thrown away and cells rinsed with 300 μL of Dulbecco’s-Phosphate Buffer Solution (D-PBS). 1.5 mL of ethanol/acetic acid mixture was used to extract the dye from cells. The quantity of extracted NR was measured by UV-visible spectrophotometry at 540 nm (Multiscan RC by Thermolab, Thermo Fisher Scientific, Helsinki, Finland). All experiments were repeated three times for each sample, using DMEM without serum (CTRL−) and latex (CTRL+) as references.

#### 2.3.2. Indirect Viability Test: 3-(4,5-Dimethylthiazol-2-yl)-2,5-diphenyltetrazolium Bromide (MTT) 

MTT is a fast colorimetric assay founded on the cleavage of a yellow tetrazolium salt to purple formazan crystals, through mitochondrial enzymes in metabolic-active cells. It is applied to appraise indirect toxicity and cell viability by spectrophotometry [34]. 96 well-cultured plates were utilized to culture cells in contact with samples extract and incubated for 24 and 72 h. Tetrazolium salt MTT (Cell Proliferation Kit II (MTT) Roche diagnostics, Indianapolis, IN, USA] was added, and after 2 h, DMSO (dimethyl sulfoxide) was also added in every well for the purpose of dissolving formazan crystals. The quantification of formazan at 540 nm is directly associated with the NAD(P)H-dependent cellular oxidoreductase enzymes in live cells. The sample extract was obtained in centrifuge tubes with a ratio between the sample and extracting solution (DMEM without bovine fetal serum) equal to 0.2 g/mL (according to ISO 10993-12) [52]. Vials were incubated at 37 °C for 72 h and then pH was measured and adjusted to have physiological cell conditions. Quantitative evaluation of yellow tetrazolium salt incorporated in cells was obtained by optical density scanning (Multiscan RC by Thermolab, ThermoFisher Scientific, Helsinki, Finland). Cells (3 × 10^3^/mL_DMEM_ for test at 24 h and 2 × 10^3^/mL_DMEM_ for tests at 72h) were seeded and incubated at 37 °C, 90% ± 5% humidity and 5.0% ± 1% CO_2_/air.

#### 2.3.3. Proliferation Test: Bromo-2-deoxyUridine (BrdU) 

BrdU test is a colorimetric immunoassay assay (Cell Proliferation ELISA, BrdU and Roche) utilized to evaluate DNA synthesis [55]. BrdU is an analogue of thymidine and can be incorporated into DNA during S-phase [56]. It can be distinguished with an antiBrdU specific antibody. Binding of the antibody requires denaturation of the DNA. BrdU labelling solution was added in cells grown in 96-well plates, and after 24 h exposure to the sample extracts, the proliferation was evaluated by absorbance at 370 nm, using the UV-visible spectrophotometer reported above. The quantification of the absorbance for the developed color is proportional to the quantity of BrdU incorporated into the cells, correlating with the newly synthesized DNA and consequently with the number of proliferating cells in the culture. Quantitative evaluation of BrdU incorporated into the newly synthetized DNA of cycling cells was obtained by optical density scanning (Multiscan RC by Thermolab, ThermoFisher Scientific, Helsinki, Finland). Cells (3 × 10^3^/mL_DMEM_ for tests at 24 h and 2 × 10^3^/mL_DMEM_ for tests at 72 h) were seeded and incubated at 37 °C, 90% ± 5% humidity and 5.0% ± 1% CO_2_/air.

### 2.4. Elemental Analysis

The compositions of the glasses were verified through Inductively Coupled Plasma Optical Emission Spectrometry (ICP-OES) with the Optima 5300 DV spectrometer (Perkin Elmer, Shelton, CT, USA). 

### 2.5. Leaching Tests

Leaching tests were performed on different replicated samples to verify the amount of cerium released during the cellular tests after the second use; the solutions were analyzed through ICP-MS Perkin Elmer Nexion 350× with autosampler ESI SeaFAST (DL = 0.0017 ppb)

### 2.6. Statistical Analyses

For all assays performed, comparisons among groups considering the first and second use samples for each glass (BG_1.2, BG_3.6 and BG_5.3), both after 24 and 72 h, were tested using Student’s *t*-test, as shown in Table 2. Differences between the first and second use samples were considered statistically significant for *p*-values <0.05 and nonsignificant for *p*-values ≥0.05. All analyses were performed using the Excel^®^ software for Windows (Microsoft Office Professional 2016, version 16.0.4266.1001, Microsoft Corporation, Redmond, WA, USA).

## 3. Results and Discussion

Cytotoxicity tests of all samples were performed using MLO-Y4 because of the well-known and documented efficacy of this cell line for in vitro studies concerning materials for bone regeneration, foreknowing the interaction between biomaterials and osteocytes [50,57].

**Figure 1:**
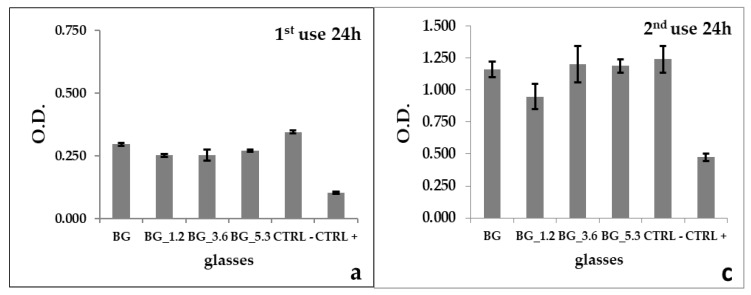

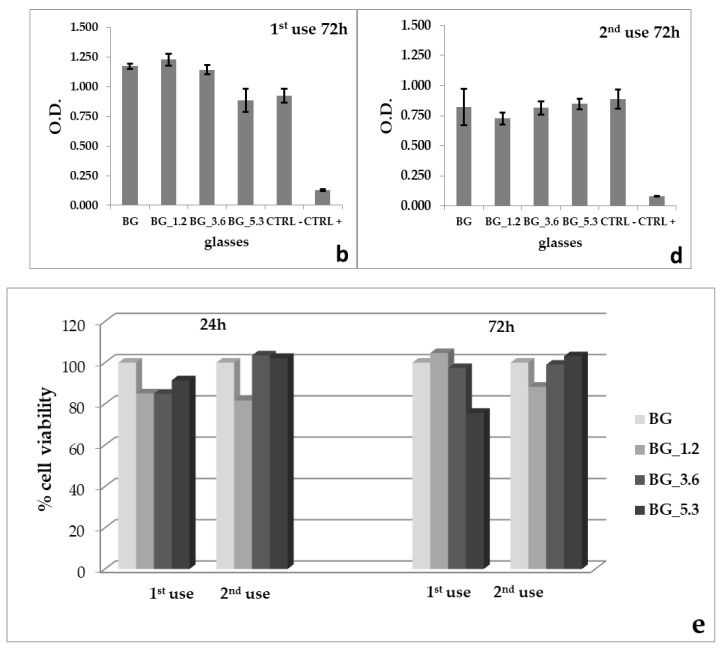
Neutral red (NR) uptake with osteocyte-like cell line murine long bone (MLO-Y4) cell lines after (**a**,**c**) 24 h, (**b**,**d**) 72 h for the parent glass BG and cerium-containing glasses BG_1.2, BG_3.6 and BG_5.3, together with negative (CTRL−) and positive (CTRL+) controls; errors bars represent standard deviation. Comparison between all doped glasses among the first and second use, after 24 and 72 h (**e**).

The results of NR uptake are reported in Figure 1a–e. In the first use, the optical density (O.D.) expressed as a percentage respect to BG (100.00%) is higher for BG_5.3 (91.43%) after 24 h, while a greater cell expansion is detected for BG_1.2 (104.66%) after 72 h. The best performance of BG_5.3 is detected after 24 h; we actually confirmed the lower NR cell uptake at a longer time (72 h) as observed in our previous paper [48].

In the second use, BG_5.3 reveals a cell viability higher than BG, both at 24 and 72 h (102.27% and 103.12%, respectively) suggesting a higher uptake and a positive effect of cerium. BG_3.6 after 24 h (103.50%) is consistent with BG_5.3, and after 72 h (99.02%), with BG. Conversely, a decrease in cell proliferation both at 24 and 72 h is observed for BG_1.2 (81.63% and 88.32%, respectively).

**Figure 2:**
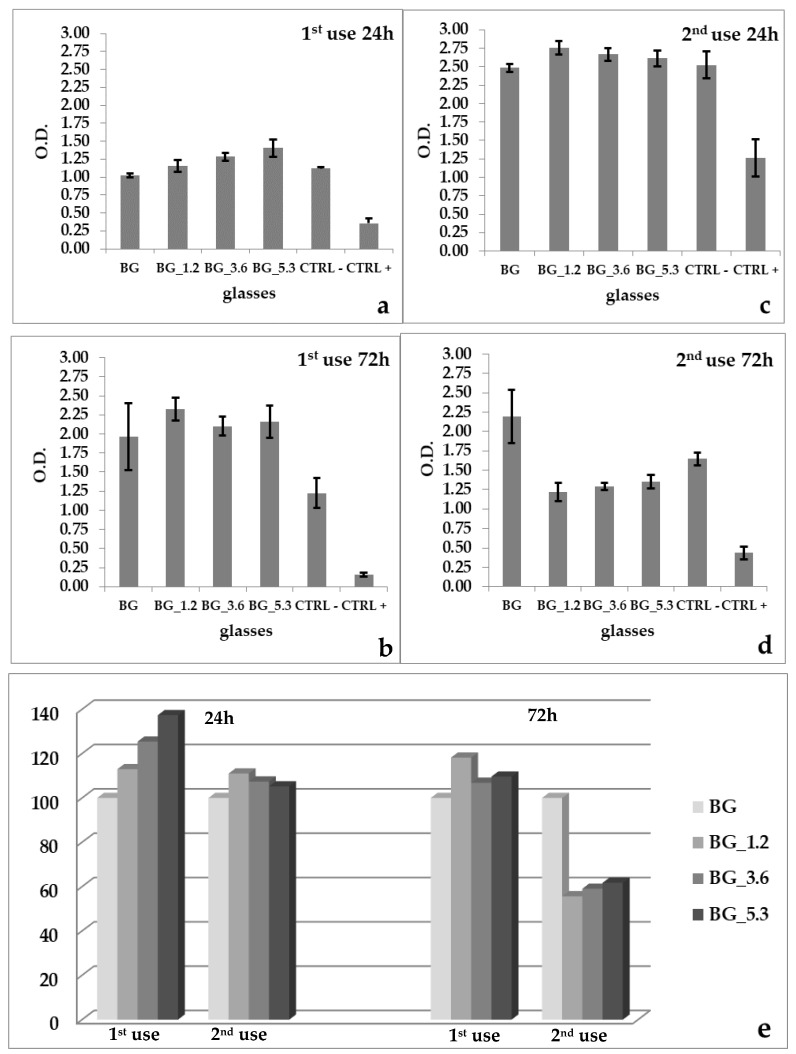
3-(4,5-Dimethylthiazol-2-yl)-2,5-diphenyltetrazolium bromide (MTT) uptake with MLOY4 cell lines after (**a**,**c**) 24 h, (**b**,**d**) 72 h for BG, BG_1.2, BG_3.6 and BG_5.3, together with negative (CTRL−) and positive (CTRL+) controls; errors bars represent standard deviation. Comparison between doped glasses among the first and second use, after 24 and 72 h (**e**).

The results of MTT tests are reported in Figure 2a–e. All cerium-doped glasses, at the first use, favor the viability of MLO-Y4 cells both after 24 h (112.98%, 125.43%, 137.36%) and 72 h (111.02%, 107.41%, 105,23%) respectively for BG_1.2, BG_3.6 and BG_5.3, as observed previously [48]. After 24 h, the results of the second use are consistent with those of the first use; on the contrary, at 72h we detected an unequivocal decrease in cell viability (55.67%, 59.06%, 61.74% respectively for BG_1.2, BG_3.6 and BG_5.3).

**Figure 3:**
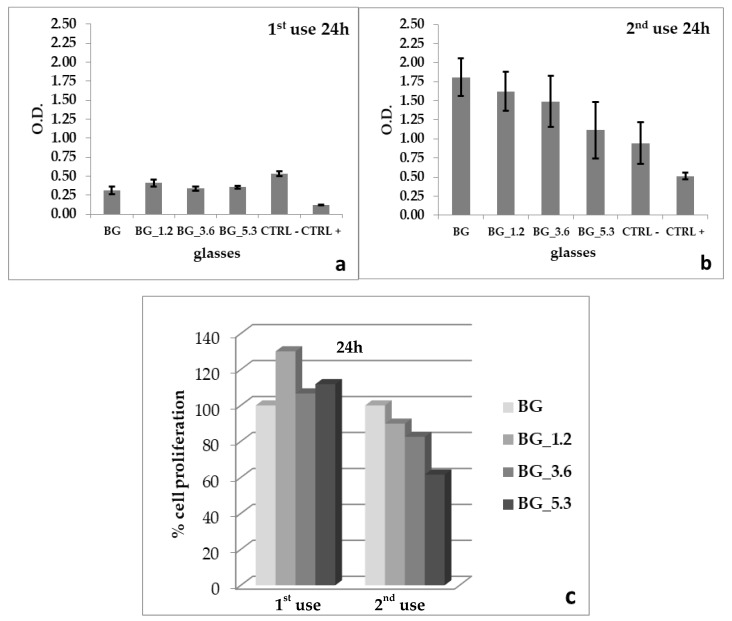
Bromo-2-deoxyUridine (BrDU) uptake with MLOY4 cell lines after (**a**,**b**) 24 h for BG, BG_1.2, BG_3.6 and BG_5.3, together with negative (CTRL−) and positive (CTRL+) controls; errors bars represent standard deviation. Comparison between doped glasses among the first and second use for 24 h (**c**).

The results of BrdU proliferation are reported in Figure 3a–c. The best performance is achieved by BG_1.2 (130.08%) at the first use; for the second use, BG_1.2 (89.85%) maintained primacy over BG_3.6 (82.51%) and BG_5.3 (61.60%).

In summary, we can assert that in the first use, the cerium-containing glasses, in comparison with BG, positively influenced the proliferation of MLOY4 cells after 24 h in MTT (112.99%–137.36%) and BrdU (106.64%–130.08%) assays; the latter was also proved in our previous study [48]. These results changed significantly in the second use; in fact, after 24 h, all doped glasses (BG_1.2, BG_3.6 and BG_5.3) gave rise to a lower cell viability (61.60%–89.85%) in comparison with BG by means of BrdU assay. Moreover, after 72 h, in the MTT assay, a marked decrease in cell proliferation in the second use (55.67%–61.74%) was observed when compared with those of the first use (109.64% to 118.22%).

We now have to ask ourselves two questions. The first question is “Are the differences among the first and second use results due to random?” and the second question is “Why is there a drop in cell proliferation in the second use results of assays based on eluate (BrdU and MTT)?”.

Student’s *t*-test results shown in Table 2 demonstrate that most of the discrepancies among the two groups are not casual, with a statistically significance *p* < 0.01 for most results. 

The reason behind the drop-in performance in the long term (72 h) and in the second use group of assays based on eluate (BrdU and MTT) needs to be argued. Important discrepancies should be expected between the findings of the direct culture tests and those arising from the indirect ones [29]. To this aim, the materials’ cytotoxicity has been investigated both through direct contact tests—where the cells are seeded directly onto the material and then incubated in suitable conditions—and indirect contact (eluate), in order to observe the possible negative effects of the samples’ eluates. 

In the second use eluates, the release of the cerium ion is < 1ppb; this is important, because in this way, we can maintain their antioxidant activity and there is not a high concentration of cerium in the body fluids of living animals or humans that can compromise their possible medical applications.

We demonstrated [48] that Ce^3+^ ions are involved in the formation of CePO_4_ after soaking in physiological fluids, and Ce^4+^ ions are probably exposed on the glass surface, capable of inducing proliferation cells, as suggested by Naganume and Traversa. [58]

Pirmohamed et al. indicated that a different amount of surface exposed to the cellular environment of the nanoceria changes the state of interaction that occurs [8]. 

On the basis of the second use results of the MTT and BrdU tests, we can suppose that the Ce^4+^ ions concentration is not sufficient in the long term to suppress cellular stress conditions by interfering with ROS generation and consequent impairment in cell proliferation. This fact seems reasonable, since in these eluates (MTT and BrdU assays) the lack of surface (in indirect test we work on filtered extracts) prevent long-term self-regenerating passage between Ce^3+^ and Ce^4+^ oxidation states, and so the capacity of these glasses to mimic CAT and SOD enzymatic-activities is low or lacking in the long-term exposure (72 h) [19]. However, it is interesting to observe that the biological performance of cerium-doped glasses at the first use is maintained in the long term (72 h) (MTT, 109.64%–118.22%), protecting cell viability from ROS derivatives. This property is no longer available in the second use after longer exposure (MTT, 55.67%–61.74%); this difference is statistically significant (*p* < 0.001), as shown in Table 2. 

On the contrary, NR uptake is based on growth analysis of cells seeded directly onto the material surface of the bioactive glass slices. After 24 h, the cells growth decreased according to NR uptake (84.89%–91.43%), but the outcomes changed after 72 h exposure (75.67%–104.66%) because cells were seeded directly onto the material. So, in NR uptake the interaction among cells and sample is slower than in BrdU and MTT: still partial after 24 h and productive after 72 h.

In NR uptake, we noticed an increase in cell proliferation in the second use for cerium doped glasses with higher cerium concentration (BG_3.6 and BG_5.3) both after 24 h (BG_3.6 103.50%; BG_5.3 102.27%) and 72 h (BG_3.6 99.02%; BG_5.3 103.11%). These data attest an enduring self-regenerating activity in the long-term, not verifiable with indirect assays. 

Thus, these bioactive glasses in direct contact with cells give rise to enhanced proliferation with respect to other materials containing ceria nanoparticles [7], both in the short and long term, even if reutilized. 

Enduring antioxidant activity of cerium doped glasses is demonstrated here by statistically significant results of cell proliferation, even in reused samples with multiple washing and cell contacts, as shown in Table 2. However, new studies in cell culture with sensitive ROS assays are needed in the future to quantify ROS activity against the self-regenerative antioxidant activity of these cerium doped bioactive glasses. 

On the basis of the present findings, a future prospective is the implantation of cerium-doped bioactive glasses in in vivo animal models as bone substitutes [59], which can be carried out with biological safety, considering that the release of the cerium ions from these bioactive glasses is very low (<1ppb). 

## 4. Conclusions

Cerium-doped glasses with a total surface of ~500 µm^2^ were studied in order to evaluate their self-regenerating antioxidant activity. For the first time, this kind of activity is studied for material with such large dimensions. 

Our previous studies indicated that these glasses are bioactive, promote cell uptake and viability and possess antioxidant properties.

New cytocompatibility tests were performed twice (first and second use) on the same bioactive glasses. The outcomes indicate that the presence of cerium ions promotes cell uptake and viability, proved by NR uptake results, in particular after 72 h, both in the first and second use group. 

On the contrary, a decrease in cell proliferation was observed in assays based on eluate (MTT and BrdU) in the second use, because the cerium ions concentration is not sufficient to overwhelm cellular stress conditions by interfering with metabolic waste. However, the low cerium release (< 1ppb) from the doped glasses allows their antioxidant activity and possible medical applications. A future prospective is their implantation as bone substitute in animal models.

## Figures and Tables

**Table 1:** Nominal composition (mol%) of the synthesized samples.

Sample	SiO_2_	Na_2_O	CaO	P_2_O_5_	CeO_2_
BG	46.2	24.3	26.9	2.6	–
BG_1.2	45.6	24.0	26.6	2.6	1.2
BG_3.6	44.5	23.4	26.0	2.5	3.6
BG_5.3	43.4	23.2	25.7	2.4	5.3

**Table 2:** Student’s *t*-test (*p* values) for the first and second use results of the cytocompatibility tests applied on the samples.

First Use vs. Second Use	NR 24 h	NR 72 h	MTT 24 h	MTT 72 h	BrdU 24 h
BG_1.2	0.25837 ^d^	0.00183 ^b^	0.68186 ^d^	0.00006 × 10^−1 a^	0.00022 ^a^
BG_3.6	0.00198 ^b^	0.76469 ^d^	0.00011 ^a^	0.00002 × 10^−2 a^	0.11442 ^d^
BG_5.3	0.00027 × 10^−1 a^	0.00014 ^a^	0.00088 ^a^	0.00002 ^a^	0.00100 ^a^

^a^*p* < 0.001, ^b^ *p* < 0.01, ^c^ *p* < 0.1, ^d^ *p* > 0.1
